# Prevalence of intestinal parasite infections and associated risk factors among patients of Jimma health center requested for stool examination, Jimma, Ethiopia

**DOI:** 10.1371/journal.pone.0247063

**Published:** 2021-02-22

**Authors:** Yohannes Alemu Belete, Tilahun Yemane Kassa, Minale Fekadie Baye

**Affiliations:** 1 School of Medical Laboratory Science, Faculty of Health Science, Institute of Health, Jimma University, Jimma, Ethiopia; 2 Department of Clinical Laboratory, School of Medical Laboratory Sciences, of Health Science, Institute of Health Jimma University, Jimma, Ethiopia; 3 Department of Biochemistry, School of Biomedical Sciences, Jimma University, Jimma, Ethiopia; National Institute for Infectious Diseases Lazzaro Spallanzani-IRCCS, ITALY

## Abstract

**Background:**

Intestinal parasitic infections are still a serious public health problem in poor and developing countries like Ethiopia. Local epidemiological data is crucial to design and monitor prevention and control strategies. This study aimed to determine the prevalence of intestinal parasite infections and associated risk factors among patients requested for stool examination at Jimma health center, Southwest, Ethiopia.

**Methods:**

A cross-sectional study was conducted among a total of 384 patients in Jimma health center, Southwest, Ethiopia. Stool samples were collected and examined using direct wet-mount and formal-ether concentration techniques. Data were analyzed using the Chi-Square (X^2^) test and SPPS Version 24 and P Value<0.05 was considered for statistically significance.

**Results:**

The overall prevalence of intestinal parasite infections was found to be 79(20.6%). The infection rate was slightly higher in females 261(68%) than in males 123(32%). Eight types of intestinal parasites were identified and the highest prevalence was *Giardia lamblia* 25(6.5%) followed by *A*. *lumbricoides* 22(5.7%). Single parasitic infection was found among 67(17.4%) of the patients and double infection was 12(3.1%). Shoe wearing habits, Status of fingernail, Handwashing before a meal and after defecation, Source of water for bathing and drinking were significant factors(p<0.05) for intestinal parasitic infection.

**Conclusion:**

A relatively low prevalence of intestinal parasite infections was observed among patients of Jimma health center requested for the stool examination.

## 1. Background

Intestinal parasites are an organism that lives in or on and takes its nourishment from other organisms. The common parasite causing Intestinal infection can be protozoa or Helminthes [1], Among helminthic parasite, *Ascaris lumbricoides*, *Trichuris trichuria*, and hookworm are the most prevalent and affect about one-sixth of the world population [2, 3].In addition to helminthic parasites protozoa parasites such as *Giardia lamblia*, *E*. *histolytica*, and cryptosporidium infections are very common in developing countries including Ethiopia and the most dominant cause of intestinal morbidity in children [4]. IPIs are among the most prevalent human parasitic infections worldwide and constitute a global health burden causing clinical morbidity and mortality [5]. According to the world health organization (WHO,2017) 3.5 billion people are infected, out of which, about 450 million are infected due to IPIs [6, 7]. The prevalence and distribution of IPIs differ from region to region due to several environmental, geographical, and social factors [8, 9].

The prevalence of IPIs would be expected higher in developing countries most likely due to low income, lack of pure water supply, inadequate sanitation, hygiene, and low level of education [10, 11]. Parasitic infections can also occur from eating contaminated raw vegetables and fruits, soil-eating behavior [12]. IPIs are most common among the poorest, school-age children, pregnant, immune-compromised patients, poor hygiene, and occupational contact with soil [13]. Like other developing countries IPIs are common in Ethiopia and cause serious public health problems such as malnutrition, anemia, and growth retardation as well as a higher susceptibility to other infections [14], and causes of outpatient morbidity in the country [15]. IPIs are the second most cause of outpatient morbidity next to malaria in Ethiopia [4]. Several studies have shown that the prevalence of IPIs was high in Ethiopia due to the lowest quality water supply and latrine coverage [16]. According to the Ethiopian Mini Demographic Health Survey, 2014 report the majority of households 89% use non-improved latrine facility and 38% of households have no toilet facility [17]. The prevalence of IPIs has been studied in different parts of the country [18–20]. These studies showed that helminthic infections represent a major public health concern. In addition to the health impact, these intestinal parasites have significant socio-economic impacts in terms of absence from work and treatment expenses [20, 21]. This study aimed to assess the prevalence of intestinal parasite infections, and associated risk factors among patients of Jimma health center requested for stool examination.

## 2. Methods

### 2.1 Study area and period

This study was conducted in Jimma health center, Southwest, Ethiopia, which is one of the public health centers found in Jimma town, southwest, Ethiopia at 353 km from Addis Ababa, the capital city of Ethiopia. The data were collected from those selected for stool examination and those who were not taken antihelminthic drugs within three months from March,06,2019 to April 06, 2019.

### 2.2. Study design

A cross-sectional study design was conducted among all Jimma health center patients who were selected for stool examination during the study period.

### 2.3. Sample size determination

The sample size (n) was determined by using a single population proportion formula [22] by considering the following assumptions. Zα/2 = critical value for normal distribution at 95% confidence level which equals 1.96 (Z value at alpha = 0.05). P = 48.25% as it was reported by the study done in Jimma [23] and d = margin of error of (0.05). The sample size was calculated as follows:

$N=\frac{{{(Z}_{\frac{\alpha }{2}}}^{2})P(1-P)}{d^{2}}$

Where,

N = sample size

P = prevalence rate

d = margin of sampling error

N = $\frac{1.96^{2})0.4825(1-0.4825)}{{\left(0.05\right)}^{2}}$ = 383.689 ~ 384

Based on the assumption the total sample size for the study was 384. The actual number of patients who participated in the study were selected using a simple random sampling technique to incorporate 384 patients.

### 2.4. Data collection and processing

The data were collected using structured self-administered questionnaires prepared in local languages (Afan Oromo and Amharic). The study participants were interviewed to obtain sex, age, educational level, Shoe wearing habits, Status of fingernail, washing habit before meal, washing habit after a meal, Hand washing after defecation, Source of water for bathing, Source of water for drinking, Latrine availability, Latrine usage, and Fruit washing before eating.

Then the responses were translated back into English by another expert fluent both in English and in local languages. The questionnaire was pre-tested on 5% of the study population, one week before starting actual data collection time, to evaluate the validity, reliability, and reaction of the study population to the test. A necessary adjustment was made accordingly before the study began. For parasitological analysis, fresh stool samples were collected. The participants were instructed properly and given clean labeled collection cups along with applicator sticks and from each participant, about 2g of fresh stool was collected. At the time of collection, the date of sampling, the name of the participant, age, sex, and education level of the participant were recorded for each subject in a recording format. The stool sample was preserved in 10% formalin before transported to the Laboratory. A 1g of each of the stool sample was processed and examined microscopically using direct wet-mount and formal-ether concentration techniques following the procedures according to WHO guidelines [24].

### 2.5. Data analysis

The data collected in the questionnaires and results collected from laboratory were entered into Epi Data version 3.1. After double data entry verification, data were exported into the Statistical Package for Social (SPSS, version 24) for analysis. Descriptive statistics were used for calculating the frequency and percentage of both dependent and independent variables. The Chi-square(X^2^) test was performed to verify the possible association between the prevalence of IPIs and variables such as sex, age, educational status, and possible risk factors. A p-value less <0.05 was declared as statistically significant.

### 2.6. Ethical considerations

Ethical Permission was obtained from Jimma University review board and Jimma town administrative officials. Written informed consent was obtained from each of the adult (age>18) participants and oral consent for minors (age<18) participants was obtained from their parents before the participation. The advantage and purpose of the study were explained to all patients and got permission from all patients who were participated in the study. The procedure of stool sample collection was not invasive and would no harm to the study subjects. The confidentiality of the result was sustained. Anti-parasitic drug prescriptions were distributed for positive cases of test and advised to take the prescribed drugs accordingly. Furthermore, health education on the transmission of intestinal parasites was given for the study population.

## 3. Results

### 3.1. Socio-demographic characteristics of study subjects

Among the 384 patients examined, 261(68%) were females, and the remaining 123(32%) males. The major age ranges of the participants were from 20 to 24 years. Regarding the educational level of the patients, illiterate 41(10.7%), read and write only 9(2.3%),grade 1–4 was 80(20.8%), grade 5–8 was 166(43.23%), grade 9–12 was 74(19.3%), and > 12 grade was 14(3.7%) "Table 1".

**Table 1:** Socio-demographic characteristics of patients examined for stool, Jimma health center, Southwest, Ethiopia (n = 384).

Socio-demographic Data	Category	Number N (%)
**Gender**	Male	123(32)
	Female	261(68)
**Age group**	0–5	20(5.2)
	6–9	21(5.4)
	10–14	24(6.25)
	15–19	53(13.8)
	20–24	90(23.44)
	25–29	69(18)
	30–34	20(5.2)
	35–39	19(4.95)
	40–44	21(5.4)
	45–49	7(1.8)
	50–54	14(3.65)
	55–59	8(2.1)
	60–64	9(2.34)
	>64	9(2.34)
**Level of education**	Illiterates	41(10.7)
	Read & write	9(2.3)
	1–4	80(20.8)
	5–8	166(43.23)
	9–12	74(19.3)
	>12	14(3.7)

### 3.2. Prevalence of intestinal parasite infections

From a total of 384 patients examined for stool, at Jimma health center, the overall prevalence of intestinal parasite infection was 79 (20.6%), Among them,23(6%) were males and 56 (14.6%) females "Table 2".

**Table 2:** Distribution of intestinal parasites prevalence respected to age and sex among patients requested for stool examination at Jimma health center, Southwest, Ethiopia (n = 384).

Age category	Male (N = 123)		Female (N = 261)		Total
	Positive N (%)	Negative N (%)	Positive N (%)	Negative N (%)	N (%)
<5	0 (0)	11(55)	1(5)	8(40)	20 (5.21)
6–9	4(19)	8(38)	2(9.52)	7(33.3)	21(5.5)
10–14	0(0)	11(45.8)	1(4.2)	12(50)	24(6.25)
15–19	1(1.9)	9(17)	9(17)	34(64.2)	53(13.8)
20–24	6(6.7)	12(13.3)	25(27.8)	47(52.2)	90(23.44)
25–29	5(7.2)	12(17.4)	9(13.04)	43(62.32)	69(18)
30–34	0(0)	3(15)	3(15)	14(70)	20(5.2)
35–39	1(5.3)	6(31.6)	1(5.3)	11(57.9)	19(4.9)
40–44	1(4.8)	7(33.3)	2(9.5)	11(52.4)	21(5.5)
45–49	0(0)	5(71.4)	0(0)	2(28.6)	7(1.8)
50–54	1(7.14)	5(35.8)	1(7.14)	7(50)	14(3.65)
55–59	2(25)	4(50)	1(12.5)	1(12.5)	8(2.1)
60–64	0(0)	5(55.6)	0(0)	4(44.4)	9(2,34)
>64	2(22.2)	2(22.2)	1(11.1)	4(44.4)	9(2.34)
Total	23(6)	100(1.6)	56(14.6)	205(53.4)	384(100)

### 3.3. Distribution of intestinal parasite prevalence and types of infections

From the total sample size (384), 79 (20.6%) intestinal parasites infected individuals were found, of these *Giardia lamblia*, was the predominant parasite with 25 infection cases (6.5%), followed by *A*.*lumbricoides* with 22 infection cases (5.7%). From 79 intestinal parasites infected individuals, 19(4.9%) males and 48(12.5%) females were single infections and 4(1%) males and 8(2%) females had double infections "Table 3".

**Table 3:** Distribution of intestinal parasite prevalence and types of infections among patients requested for stool examination, Jimma health center, Southwest, Ethiopia (n = 384).

Parasites identified	Male N (%)	Female N (%)	Total N (%)	Single infection		Double infection	
				M	F	Male	Female
				N (%)	N (%)	N (%)	N (%)
***A*.*lumbricoides***	7(1.8)	15(3.9)	22(5.7)	7(1.8)	13(3.4)	1(0.3)	1(0.3)
***G*. *intestinalis***	5(1.3)	20(5.2)	25(6.5)	4(1)	17(4.4)	0(0.0)	4(1)
***E*.*histolytica/dispar***	3(0.8)	13(3.4)	16(4.2)	3(0.8)	12(3.1)	0(0.0)	1(0.3)
***Taenia species***	2(0.5)	3(0.8)	5(1.3)	2(0.5)	3(0.8)	0(0.0)	0(0.0)
***Hookworm***	1(0.3)	2(0.5)	3(0.8)	0(0.0)	2(0.5)	1(0.3)	0(0.0)
***T*. *trichuria***	2(0.5)	2(0.5)	4(1)	0(0.0)	0(0.0)	2(0.5)	2(0.5)
***H*. *nana***	2(0.5)	0(0.0)	2(0.5)	2(0.5)	0(0.0)	0(0.0)	0(0.0)
***S*. *mansoni***	1(0.3)	1(0.3)	2(0.5)	1(0.3)	1(0.3)	0(0.0)	0(0.0)
**Total**	23(6)	56(14.6)	79(100)	19(4.9)	48(12.5)	4(1)	8(2.1)

### 3.4. Association of different risk factors with intestinal parasitic infections

Based on the chi-square(X^2)^ test statistically significant associations were shown between the prevalence of IPIs and the risk factors including participants shoe wearing habits (X^2^ = 6.3622, P = 0.04154), Status of a fingernail (X^2^ = 66.2318, P = 0.00001), hand washing before a meal (X^2^ = 6.2553, P = 0.04382), hand washing after defecation(X^2 =^ 15.7892, P = 0.000373), Source of water for bathing (X^2^_=_ 15.7603, P = 0.000378)and Source of water for drinking (X^2 =^ 5.4411, P = 0.019668) were significantly associated. However, prevalence of IPIs was not associated with sex, educational status, latrine availability, latrine usage, and fruit washing before eating "Table 4".

**Table 4:** Chi- square analysis, factors associated with the prevalence of intestinal parasitic infections among patients of Jimma health center, Southwest, Ethiopia.

Variables	Categories	Parasitic Infection		x^2^	p-value
		Positive N (%)	Negative N (%)		
**Sex**	Male	23(18.7)	100(81.3)	0.3888	0.532922
	Female	56(24.5)	205(78.5)		
**Educational status**	Illiterate	9(18)	41(82%)	3.8911	0.420944
	1–4	17(21.25)	63(78.75)		
	5–8	40(24.1)	126(75.9)		
	9–12	12(16.2)	62(84.8)		
	>12	1(7.1)	13(92.85)		
**Shoe wearing habits**	Always	64(18.8)	277(81.2)	6.3622	**0.04154**^**a**^
	Sometimes	14(34.1)	27(65.85)		
	Not at all	1(50)	1(50%)		
**Status of fingernail**	Trimmed	56(15.7)	299(84.2)	66.2318	**0.00001**^**a**^
	Not trimmed	23(79.3)	6(20.7)		
**Hand washing before a meal**	Always	3(25)	9(75)	6.2553	**0.04382**^**a**^
	Sometimes	21(14.1)	128		
	Not at all	55(24.7)	168(75.3)		
**Hand washing after defecation**	Always	5(26.3)	14(73.7)	15.7892	**0.000373**^**a**^
	Sometimes	30(13.6)	191(86.4)		
	Not at all	44(30.6)	100(69.4)		
**Source of water for bathing**	Well	5(83.3)	1(16.7)	15.7603	**0.000378**^**a**^
	Spring	3(33.3)	6(66.7)		
	Pipe	71(19.2)	298(80.7)		
**Source of water for drinking**	Pipe	74(19.8)	300(80.2)	5.4411	**0.019668**^**a**^
	Spring	5(50)	5(50)		
**Latrine availability**	Yes	78(20.4)	304(79.6)	1.0654	0.301978
	No	1(50)	1(50)		
**Latrine usage**	Always	71(20)	284(80)	1.9792	0.159471
	Sometimes	9(31)	20(69)		
**Fruit washing before eating**	Always	5(29.4)	12(70.6)	5.7734	0.05576
	Sometimes	71(19.6)	291(80.4)		
	Not at all	3(60)	2(40)		

^a^ = statistically significant (p<0.05).

## 4. Discussion

The current study finding indicated that the overall prevalence of IPIs among Jimma health center suspected patients were found to be 20.6%. This finding is lower than a study conducted in different areas of Ethiopia, namely, in Jimma town 48.2% [23], 37%) [25], and 89.7% [26] in Wonji showa 24.3% [27], in Hawasa 26.6% [28], and in Gondar (34.2%) [29] and in Northwest Ethiopia 31.5% [30]. However, the prevalence of IPIs in the current study, was higher than studies conducted in Ghana 17.33% [31] and Brazil 17.5% [32]. The differences in finding among a range of studies could be explained by the methods employed for stool examination, diversity of health condition, water supply, feeding habit, cultural practices in the different study area, the study period, age variations, and geographical differences may have also contributed to the differences.

*Ascaris lumbricoides* was the predominant helminthic parasitic infection in study 5.7%. This finding was lower than that of a similar study reported in Jimma 14.6% [25], in Gonder 5.9% [29], and Abaye Deneba 8.4% [33]. However, this finding is higher than that of a study conducted in Brazil 1.6% [32].

The most prevalence protozoan parasitic infection in the current study was *Giardia lamblia* 6.5% which was comparable with a study done at Mojo health center 6.5% [14], and Hawasa 7% [28], but lower than a study done at Felege Hiwot referral hospital 13.3% [34]. This might be due to differences in water supply, feeding habits, environmental sanitation, and awareness of the ways of transmission and prevention and control measures of this parasitic infection. The age group of 20–24 was the most affected. This might be due to occupationally related exposures of this age group. Regarding sex, the prevalence of intestinal parasite infection was relatively higher in females than males. This result is agreed with studies carried out in Gondar [35] and Southwest of Iran [36]. In contrast to this finding, previous studies carried out in Azezo North-western Ethiopia [37], in Bahirdar [38], in Nepal [39], and in Brazil [40] indicated that males were at higher risk of having IPIs than females. This variation of exposure among the different sex groups might be due to differences in occupational exposure in different communities and study area.

Single infection occurred in 67 individuals making 17% of the total examined patients. Of these most females 48(12.5%) had a single infection. This finding was comparable to the study done in Gondar [35] and Mota Town [41]. The level of environmental sanitation, source of water, poor personal hygiene, and individual behavioural and personal condition are very important risk factors for intestinal parasite infection. The current finding showed IPIs was associated with shoe wearing habits, Status of a fingernail, handwashing habit before a meal, handwashing habit after defecation, and Source of water for drinking and bathing. The present study was agreed with the previous studies reported in Gondar [35], in Mota Town [41] in Delgi school North Gondar [42], and in Arbaminch Southern Ethiopia [43]. On the contrary, latrine availability, latrine usage, and fruit washing before eating were not associated with the prevalence of IPIs.This finding was contradicted the study in Jawi town, north -west Ethiopia [44], in Chencha town southern Ethiopia [45], and Mota Town [41]. The contradictory reports could be due to the category of the study population, the period of the study, and the methods employed for stool examination.

## 5. Conclusion

The present study revealed that relatively low prevalence of intestinal parasite infections was observed among patients of Jimma health center requested for the stool exam in the study period. *Giardia lamblia* was the most common parasite isolated, followed by *Ascaris lumbricoides* and *Entamoeba histolytica*. A significant relationship was observed between intestinal parasite infection with water source for both drinking and bathing, handwashing before meal and after defecation, status of fingernail and shoe wearing habit. This study has, therefore, provides baseline information for future studies and investigation on important risk factors for intestinal parasite infection in the study area. Further measures including water supply and treatment, personal and environmental hygiene, improving health education should be taken into account. Furthermore, all stakeholders should be given attention to raise awareness about control of IPIs, personal and environmental hygiene, and improving the quality of drinking and bathing water sources.

### Limitation

The major limitation of this study is that prevalence of IPIs was determined by examination of single stool specimens from each study participant. Thus, we could not access the intra- and inter stool variation of egg output. Furthermore, a single saline wet mount and formol- ether concentration technique was examined for each of the stool specimens that may affect the accuracy of the egg count.

## Abbreviations

AIDS: Acquired Immune Deficiency Syndrome
ART: Anti-Retroviral Therapy
HIV: Human Immune Deficiency Virus
IPIs: Intestinal Parasitic Infections
IRB: Institutional review board
NGO: Non-governmental organization
SAF: Sodium-acetate-Acetic acid Formalin
Spps: Species
SPSS: statistical package for social sciences
STHI: Soil-Transmitted Helminthic Infection
WHO: World Health Organization

## References

[1] Tadesse G: The prevalence of intestinal helminthic infections and associated risk factors among school children in Babile town, eastern Ethiopia. *Ethiopian Journal of Health Development* 2005, 19(2):140–147.

[2] Stephenson LS, Latham MC, Kinoti SN, Kurz KM, Brigham HJTotRSoTM, Hygiene: Improvements in physical fitness of Kenyan schoolboys infected with hookworm, Trichuris trichiura and Ascaris lumbricoides following a single dose of albendazole. 1990, 84(2):277–282. 10.1016/0035-9203(90)90286-n 2389321

[3] Organization WH: Assessing the efficacy of anthelminthic drugs against schistosomiasis and soil-transmitted helminthiases. 2013.

[4] Merid Y, Hegazy M, Mekete G, Teklemariam SJTEJoHD: Intestinal helminthic infection among children at Lake Awassa area, South Ethiopia. 2001, 15(1).

[5] Quihui L, Valencia ME, Crompton DW, Phillips S, Hagan P, Morales G, et al: Role of the employment status and education of mothers in the prevalence of intestinal parasitic infections in Mexican rural schoolchildren. *BMC Public Health* 2006, 6(1):225 10.1186/1471-2458-6-225 16956417 PMC1584408

[6] Organization WH: World Health Report. Conquering Suffering Enriching Humanity. Geneva: WHO; 1997. Available from: apps who int/bookorders/MDIbookPDF/Book/12401997 pdf [Accessed on 22 July 2017].

[7] Bahmani P, Maleki A, Sadeghi S, Shahmoradi B, Ghahremani E: Prevalence of intestinal protozoa infections and associated risk factors among schoolchildren in Sanandaj City, Iran. *Iranian journal of parasitology* 2017, 12(1):108 28761467 PMC5522686

[8] Abossie A, Seid MJBph: Assessment of the prevalence of intestinal parasitosis and associated risk factors among primary school children in Chencha town, Southern Ethiopia. 2014, 14(1):166. 10.1186/1471-2458-14-166 24528627 PMC3933408

[9] Reji P, Belay G, Erko B, Legesse M, Belay MJAjophc, medicine f: Intestinal parasitic infections and malnutrition amongst first-cycle primary schoolchildren in Adama, Ethiopia. 2011, 3(1).

[10] Kang G, Mathew MS, Prasanna Rajan D, Daniel JD, Mathan MM, Mathan V, et al: Prevalence of intestinal parasites in rural Southern Indians. *Tropical Medicine & International Health* 1998, 3(1):70–75. 10.1046/j.1365-3156.1998.00175.x 9484973

[11] Yimam YT, Gelaye KA, Chercos DHJTPAmj: Latrine utilization and associated factors among people living in rural areas of Denbia district, Northwest Ethiopia, 2013, a cross-sectional study. 2014, 18. 10.11604/pamj.2014.18.334.4206 PMC4250030 25478055

[12] Bekele F, Tefera T, Biresaw G, Yohannes T: Parasitic contamination of raw vegetables and fruits collected from selected local markets in Arba Minch town, Southern Ethiopia. *Infectious diseases of poverty* 2017, 6(1):19 10.1186/s40249-016-0226-6 28264707 PMC5340032

[13] Omrani VF, Fallahi S, Rostami A, Siyadatpanah A, Barzgarpour G, Mehravar S, et al: Prevalence of intestinal parasite infections and associated clinical symptoms among patients with end-stage renal disease undergoing hemodialysis. *Infection* 2015, 43(5):537–544. 10.1007/s15010-015-0778-6 25869822

[14] Chala B: Prevalence of intestinal parasitic infections in Mojo Health Center, Eastern Ethiopia: a 6-year (2005–2010) retrospective Study. *Epidemiol* 2013, 3(119):2161–1165.1000119.

[15] Mengistu A, Gebre-Selassie S, Kassa T: Prevalence of intestinal parasitic infections among urban dwellers in southwest Ethiopia. *Ethiopian Journal of Health Development* 2007, 21(1):12–17.

[16] Alemu A, Atnafu A, Addis Z, Shiferaw Y, Teklu T, Mathewos B, et al: Soil transmitted helminths and Schistosoma mansoni infections among school children in Zarima town, northwest Ethiopia. 2011, 11(1):189. 10.1186/1471-2334-11-189 PMC3142518 21740589

[17] Agency CS: Ethiopia mini demographic and health survey 2014. In.: CSA Addis Ababa; 2014.

[18] Dejene TJEJoHS: Impact of irrigation on the prevalence of intestinal parasite infections with emphasis on schistosomiasis in Hintallo-Wejerat, North Ethiopia. 2008, 18(2).

[19] Mengistu A, Gebre-Selassie S, Kassa TJEJoHD: Prevalence of intestinal parasitic infections among urban dwellers in southwest Ethiopia. 2007, 21(1):12–17.

[20] Beyene G, Tasew HJAocm, antimicrobials: Prevalence of intestinal parasite, Shigella and Salmonella species among diarrheal children in Jimma health center, Jimma southwest Ethiopia: a cross sectional study. 2014, 13(1):10 10.1186/1476-0711-13-10 24499189 PMC3922032

[21] Wegayehu T, Tsalla T, Seifu B, Teklu T: Prevalence of intestinal parasitic infections among highland and lowland dwellers in Gamo area, South Ethiopia. *BMC Public Health* 2013, 13(1):151 10.1186/1471-2458-13-151 23419037 PMC3584849

[22] Daniel WJNY: Biostatistics: A Foundation for analysis in the health sciences, 7th ed R Wiley 1999.

[23] Jejaw A, Zeynudin A, Zemene E, Belay T: Status of intestinal parasitic infections among residents of Jimma Town, Ethiopia. *BMC research notes* 2014, 7(1):502 10.1186/1756-0500-7-502 25100301 PMC4266909

[24] Organization WH: Basic laboratory methods in medical parasitology. Basic laboratory methods in medical parasitology 1992.

[25] Tadesse G, Zeynudin A, Mekonnen Z, Taha M, Adamu H, Kebede A: Intestinal Parasitosis among HIV Sero Positive in Jimma, Ethiopia. *J Trop Dis* 2013, 1(122):2.

[26] Lakew A, Kibru G, Biruksew A: Prevalence of intestinal parasites among street beggars in Jimma town, Southwest Ethiopia. *Asian Pacific Journal of Tropical Disease* 2015, 5:S85–S88.

[27] Degarege A, Erko B: Prevalence of intestinal parasitic infections among children under five years of age with emphasis on Schistosoma mansoni in Wonji Shoa Sugar Estate, Ethiopia. *PLoS One* 2014, 9(10). 10.1371/journal.pone.0109793 25296337 PMC4190315

[28] Mulatu G, Zeynudin A, Zemene E, Debalke S, Beyene G: Intestinal parasitic infections among children under five years of age presenting with diarrhoeal diseases to two public health facilities in Hawassa, South Ethiopia. *Infectious diseases of poverty* 2015, 4(1):49. 26530964 10.1186/s40249-015-0081-x PMC4632267

[29] Gelaw A, Anagaw B, Nigussie B, Silesh B, Yirga A, Alem M, et al: Prevalence of intestinal parasitic infections and risk factors among schoolchildren at the University of Gondar Community School, Northwest Ethiopia: a cross-sectional study. *BMC public health* 2013, 13(1):304 10.1186/1471-2458-13-304 23560704 PMC3621079

[30] Derso A, Nibret E, Munshea A: Prevalence of intestinal parasitic infections and associated risk factors among pregnant women attending antenatal care center at Felege Hiwot Referral Hospital, northwest Ethiopia. *BMC infectious diseases* 2016, 16(1):530 10.1186/s12879-016-1859-6 27716099 PMC5045606

[31] Mirisho R: Intestinal Helminths Infestation in Children Attending Princess Marie Louise Children’s Hospital. University of Ghana; 2015. 10.1155/2017/8524985 PMC5605797 29057116

[32] Faria CP, Zanini GM, Dias GS, da Silva S, de Freitas MB, Almendra R, et al: Geospatial distribution of intestinal parasitic infections in Rio de Janeiro (Brazil) and its association with social determinants. *PLoS neglected tropical diseases* 2017, 11(3):e0005445 10.1371/journal.pntd.0005445 28273080 PMC5358884

[33] Nyantekyi L, Legesse M, Medhin G, Animut A, Tadesse K, Macias C, et al: Community awareness of intestinal parasites and the prevalence of infection among community members of rural Abaye Deneba area, Ethiopia. *Asian Pacific journal of tropical biomedicine* 2014, 4:S152–S157. 10.12980/APJTB.4.2014C764 25183071 PMC4025342

[34] Derso A, Nibret E, Munshea AJBid: Prevalence of intestinal parasitic infections and associated risk factors among pregnant women attending antenatal care center at Felege Hiwot Referral Hospital, northwest Ethiopia. 2016, 16(1):530. 10.1186/s12879-016-1859-6 PMC5045606 27716099

[35] Gelaw A, Anagaw B, Nigussie B, Silesh B, Yirga A, Alem M, et al: Prevalence of intestinal parasitic infections and risk factors among schoolchildren at the University of Gondar Community School, Northwest Ethiopia: a cross-sectional study. 2013, 13(1):304. 10.1186/1471-2458-13-304 PMC3621079 23560704

[36] Fallahizadeh S, Feiz-Haddad MH, Kazemi F, Afrisham R: Prevalence of Intestinal Parasitic Infections in Shush County, Southwest of Iran during 2014–2016. *International Journal of Infection* 2017, 4(3):e14588.

[37] Endris M, Lemma W, Belyhun Y, Moges B, Gelaw A, Angaw BJEJHBS: Prevalence of intestinal parasites and associated risk factors among students of Atse Fasil general elementary school Azezo, Northwestern Ethiopia. 2010, 3(1):25–33.

[38] Hailegebriel TJBid: Prevalence of intestinal parasitic infections and associated risk factors among students at Dona Berber primary school, Bahir Dar, Ethiopia. 2017, 17(1):362. 10.1186/s12879-017-2466-x PMC5442677 28535750

[39] Khanal L, Choudhury D, Rai S, Sapkota J, Barakoti A, Amatya R, et al: Prevalence of intestinal worm infestations among school children in Kathmandu, Nepal. 2011, 13(4):272–274. 23016478

[40] Faria CP, Zanini GM, Dias GS, da Silva S, de Freitas MB, Almendra R, et al: Geospatial distribution of intestinal parasitic infections in Rio de Janeiro (Brazil) and its association with social determinants. 2017, 11(3):e0005445. 10.1371/journal.pntd.0005445 PMC5358884 28273080

[41] Asemahagn MAJAJoPHR: Parasitic infection and associated factors among the primary school children in Motta town, western Amhara, Ethiopia. 2014, 2(6):248–254. 10.1186/1472-6963-14-431 25253270 PMC4263061

[42] Ayalew A, Debebe T, Worku AJJoP, Biology V: Prevalence and risk factors of intestinal parasites among Delgi school children, North Gondar, Ethiopia. 2011, 3(5):75–81.

[43] Alemu G, Aschalew Z, Zerihun EJBid: Burden of intestinal helminths and associated factors three years after initiation of mass drug administration in Arbaminch Zuria district, southern Ethiopia. 2018, 18(1):435. 10.1186/s12879-018-3330-3 PMC6114701 30157789

[44] Sitotaw B, Mekuriaw H, Damtie DJBid: Prevalence of intestinal parasitic infections and associated risk factors among Jawi primary school children, Jawi town, north-west Ethiopia. 2019, 19(1):341. 10.1186/s12879-019-3971-x PMC6485161 31023271

[45] Abossie A, Sied MJBPH: Assessments of the prevalence of intestinal parasitic infection and associated risk factors among primary school children in Chencha town southern Ethiopia. 2014, 14(166):47–58. 10.1186/1471-2458-14-166 24528627 PMC3933408

